# Development of Gelled-Oil Nanoparticles for the Encapsulation and Release of Berberine

**DOI:** 10.1021/acsomega.3c04230

**Published:** 2023-09-06

**Authors:** Darren A. Makeiff, Brad Smith, Khalid Azyat, Mike Xia, Syed Benazir Alam

**Affiliations:** Nanotechnology Research Center, National Research Council of Canada, 11421 Saskatchewan Drive, Edmonton, Alberta T6G 2M9, Canada

## Abstract

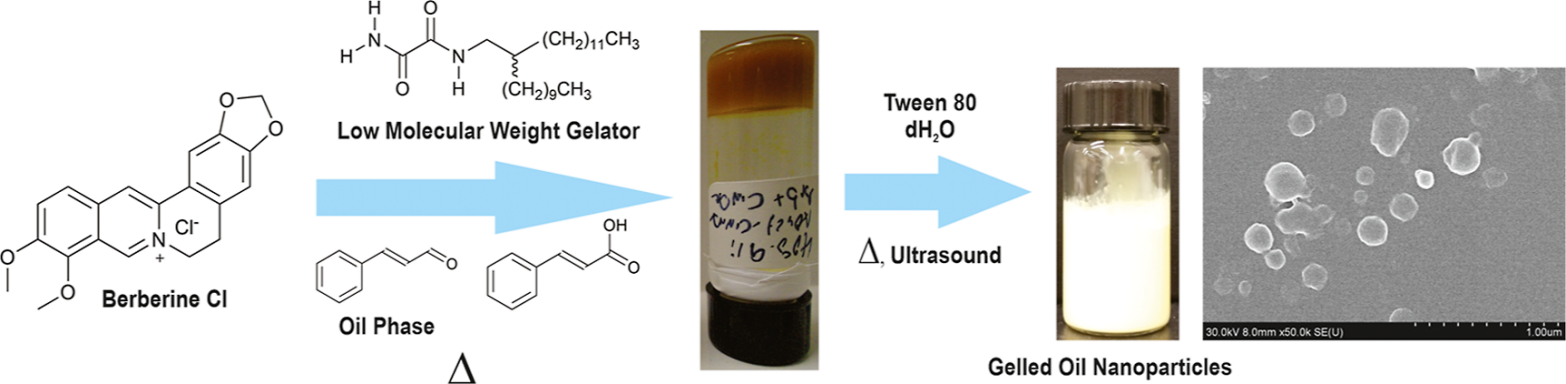

In this study, a new drug carrier based on gelled-oil nanoparticles (GNPs) was designed and synthesized for the encapsulation and release of the model hydrophobic drug, berberine chloride (BCl). Two compositions with different oil phases were examined, sesame oil (SO) and cinnamaldehyde (Cin), which were emulsified with water, stabilized with Tween 80 (Tw80), and gelled using an N-alkylated primary oxalamide low-molecular-weight gelator (LMWG) to give stable dispersions of GNPs between 100 and 200 nm in size. The GNP formulation with Cin was significantly favored over SO due to (1) lower gel melting temperatures, (2) higher gel mechanical strength, and (3) significantly higher solubility, encapsulation efficiency, and loading of BCl. Also, the solubility and loading of BCl in Cin were significantly increased (at least 7-fold) with the addition of cinnamic acid. In vitro release studies showed that the release of BCl from the GNPs was independent of gelator concentration and lower than that for BCl solution and the corresponding nanoemulsion (no LWMG). Also, cell internalization studies suggested that the N-alkylated primary oxalamide LMWG did not interfere with the internalization efficiency of BCl into mouse mast cells. Altogether, this work demonstrates the potential use of these new GNP formulations for biomedical studies involving the encapsulation of drugs and nutraceuticals and their controlled release.

## Introduction

Berberine is a well-known isoquinoline alkaloid that has been used in Chinese medicine for thousands of years and is present in the root, stem, bark, and bitter fruits of various plant and tree sources, such as *Berberis* species (spp), *Berberidaceae* spp, *Thalictrum* spp, *Chelidonium* spp, *Hydrostis* spp, *Phellodendron* spp, *Phellodendron amurense*, *Coptis chinesis*, *Hydrastis canadensis,* and others.^1–3^ Berberine has known antimicrobial, antioxidant, anti-inflammatory, antidiabetic, antiviral, antifungal, and anticancer pharmacological properties in addition to cholesterol lowering, neuroprotection, hypoglycemic, and hepato and chemoprotective properties^2^ and thus has been clinically proven for the treatment of a variety of diseases including diarrhea, ulcers, diabetes mellitus, cardiovascular, hypercholesterolemia, fatty liver, polycystic ovary syndrome, as well as a variety of different types of cancers.^1–3^ The most common form of berberine is the chloride salt BCl, which has low solubility in water (∼1.3 mg/mL).^4^ As a result, BCl has limited bioavailability, a low absorption rate, and is susceptible to metabolic degradation in the tissues, which poses serious challenges in the delivery to target tissue sites.^1–5^

Nanoencapsulation is an effective strategy for improving the efficacy, specificity, and targeting ability of therapeutic molecules with low water solubility.^6–8^ Nanosized carrier systems protect their payload from premature degradation in biological environments, enhance bioavailability, and prolong their presence in blood and cellular uptake.^8^ Types of nanocarriers include emulsions,^9^ lipid-based nanoparticles (solid lipid nanoparticles, liposomes, lipid nanoparticles),^10,11^ micelles,^12^ reversed micelles,^13^ vesicles,^14^ metal nanoparticles,^15^ mesoporous silica nanoparticles,^16^ polymer nanoparticles,^17^ dendrimers,^18^ as well as others.^19^ However, despite the wide range of reported nanocarrier systems, few have been approved by the FDA. Furthermore, designing novel drug delivery systems with considerable physical and chemical stability, high encapsulation efficiency, and loading capacity, as well as favorable release properties remains a significant challenge.

Gels from low-molecular-weight gelators (LMWGs) are fascinating pseudosolid, viscoelastic soft materials with a variety of different properties^20,21^ for numerous biomedical applications^22^ including drug delivery.^23^ LMWGs undergo reversible, hierarchical self-assembly to mostly form one-dimensional aggregates or nanofibers that can further entangle to form a volume spanning self-assembled fibrillar network (SAFIN).^20,21^ The process can be reversibly triggered by a variety of different stimuli, but most commonly heat, and is driven by the formation of noncovalent interactions such as hydrogen bonding, π–π stacking, and van der Waals and metal–ligand interactions.^20,21^ Bulk organogels from LMWGs can be easily downsized to aqueous colloidal dispersions of gelled-oil nanoparticles (GNPs) via a two-step process in which emulsified oil droplets are typically generated via a hot emulsification process followed by cooling.^24–27^ Although GNPs have exhibited the potential for the encapsulation, protection, and delivery of a variety of bioactives with low water solubility (i.e., nile red and efavirenz,^28^ rose bengal and hypericine,^29^ rhodamine 123,^30^ curcumin,^31,32^ curcuminaldehyde,^33^ sunscreen,^34^ indomethacin and ketoconazole,^35^ metallophthalocyanine,^36^ flurbiprofen,^37^ β-carotene,^38^ coumarin,^39^ paclitaxel,^40,41^ and doxorubicin^42^), they are still relatively seldom used. The commercially available 12-hydroxystearic acid (HSA) is the most commonly used LMWG for GNPs,^25,28,34–37,40,41^ and thus the development of new custom LWMGs offers the opportunity to form gels with a wider variety of organic liquids^43,44^ as well as introduce other interesting properties to trigger different types of stimuli-responsive systems that could enable tunable release characteristics.^45–47^

Recently, we reported a family of versatile and efficient LMWGs based on N-alkylated primary oxalamides (i.e., **AOx24**, Figure S1) for a variety of different organic solvents, which were also biocompatible with mouse mast cells.^48^ Coupled with the known health benefits and low aqueous solubility of BCl, the objective of the current study was to develop formulations of BCl-loaded GNPs from Tw80-stabilized nanoemulsions and a LMWG, in order to achieve a high BCl loading. Sesame oil (SO) and cinnamaldehyde (Cin) were the oils selected for this study, which are both generally regarded as safe (GRAS) by the Food and Drug Administration (FDA)^49^ and are used in many foods. The properties of the organogels from **AOx24** with both oils were characterized in terms of the critical gelator concentration (CGC), thermal stability, viscoelastic properties, and morphology. GNPs using both SO and Cin, stabilized by Tween 80 (Tw80) were prepared by hot mixing using ultrasonication and were characterized using dynamic light scattering (DLS), optical microscopy (OM), fluorescence microscopy, and scanning electron microscopy (SEM). A method to improve the solubility of BCl and loading into GNPs is discussed, in addition to the release of BCl from the GNPs into aqueous recipient media and cell internalization studies involving bone marrow-derived mouse mast cells (BMMCs).

## Experimental Section

### Materials and Methods

All reagents and solvents were commercially available and used without purification, except for the organogelator **AOx24**, which was synthesized according to a published procedure. Sesame oil was obtained from Fisher Scientific Company (Ottawa, ON, CAN). Cin (natural, ≥95%), *trans*-Cin (99%), CA, and Tw80 were obtained from Sigma-Aldrich (Mississauga, ON, Canada). Phosphate buffered saline (PBS), 10× solution, was obtained from Fisher Scientific. Water was purified using a Millipore Milli-Q Biocel System (Millipore Sigma, St. Louis, MO, USA) with a minimum resistivity of 18.2 MΩ cm. Differential scanning calorimetry (DSC) was carried out using a Q2000 DSC instrument (TA Instruments). Rheology experiments were carried out using a Discovery HR-3 rheometer (TA Instruments, New Castle, DE, USA) equipped with a parallel plate geometry (25 mm diameter and 0.5 mm gap) and a Peltier system for temperature control. Optical microscopy (OM) and polarized optical microscopy (POM) images were acquired using a Zeiss Axio Scope A1 instrument in different contrast and polarization modes. Dynamic light scattering (DLS) measurements were carried out using a Zetasizer Nano ZS (Malvern Instruments, Malvern, Worcestershire, UK) at 25 °C with polystyrene cuvettes at a scattering angle of 173°. Fluorescence microscopy images were acquired using an inverted fluorescence microscope (IX81, Olympus Canada Inc., Canada) equipped with a Fluorescein isothiocyanate (FITC) filter. Scanning electron microscopy (SEM) and scanning transmission electron microscopy (STEM) images were acquired using a cold field emission scanning electron microscope (Hitachi S4800, Tokyo, Japan). High-performance liquid chromatography (HPLC) was carried out using an Agilent 1100 HPLC (Agilent, Santa Clara, CA, USA) equipped with a diode-array detector and a ZORBAX StableBond 80 Å C18, 4.6 mm × 250 mm, 5 μm HPLC column. Flow cytometry was carried out using a CytoFLEX flow cytometer (Beckman Coulter, Brea, CA, USA) equipped with an argon ion laser (488–514 nm) and a bandpass filter to enable detection fluorescence emission at 516 nm. 20,000 events per sample were acquired at a flow rate of 30 μL/min at room temperature.

### Synthesis of 2-Decyltetradecyl-*N*-oxalamide (AOx24)

The LMWG **AOx24** was synthesized using a recently published procedure.^48^

### Organogel Preparation, Gelation Behavior, and Characterization

The powder of **AOx24** was dissolved in organic liquids with heating until clear solutions were obtained followed by cooling to 23 °C. After a period of time (typically between 0.5 and 3 h), organogel formation was evaluated using the “vial inversion” test.^50^ The mixture was classified as a gel if the material did not flow and was able to support its own weight under the influence of gravity for at least 30 s.

### Critical Gelation Concentrations

The CGCs were determined by carrying out a series of vial inversion tests at different gelator concentrations, which typically involved gelator increments of 1–2 mg/mL (or 0.1–0.2 wt %). The CGC was the lowest gelator concentration at which the sample did not flow or fall at 23 °C.

### Thermal Stability

The gel-to-sol transition temperatures (*T*_gel_) were determined using oscillatory rheology, DSC, and benchtop rheology (“vial inversion”) methods.^50^

### Rheology

The organogel samples were transferred to the rheometer as solutions heated above the gel-to-sol transition temperature (80 °C) and then cooled to 23 °C for at least 15 min prior to measurement to allow for gel formation. Oscillatory strain sweeps (0.1–100%) were carried out at a fixed frequency of 1 Hz. Angular frequency sweep experiments (0.1–1000 Hz) were carried out at a fixed strain of 0.015%. Temperature sweep experiments were carried out between 23 and 80 °C at a heating rate of 5 °C/min and fixed frequency and strain values of 1 Hz and 0.15%, respectively. The solid-to-liquid transition temperature (*T*_gel_) is the temperature at which *G*′ and *G*″ crossover (i.e., *G*′ = *G*″).

### Differential Scanning Calorimetry

Preformed gel samples were analyzed by DSC in hermetically sealed aluminum pans between 20 and 100 °C at heating and cooling rates of 10 °C/min.

### Benchtop Rheology (“Vial Inversion”) Method

Organogels from **AOx24** with solvent in capped glass vials sealed with Teflon tape were heated in an oven with a window at a rate of 1–2 °C/min. The temperature at which the gel fell to the bottom of the inverted vial was reported as *T*_gel_.

### Hansen Solubility Parameters

Solubility tests were carried out for berberine chloride (BCl) with 21 common solvents with known Hansen solubility parameter (HSP) values obtained from the literature (Table S1).^51,52^ BCl (1 and 5 mg) was mixed with each solvent (1 mL) with heating, followed by cooling the mixture to 23 °C. If a clear solution was formed, the test result was labeled S (1). If the solid did not completely dissolve, then the test result was labeled I (0). If the solid did completely dissolve but then precipitated upon cooling, the test result was also labeled I (0). The HSPs for BCl (Table S2) were determined using the HSPs in Practice (HSPiP) software.^51,52^ Using the HSPs for BCl (Table S2), the affinity of BCl and a solvent can be predicted if the HSPs for the solvent are known by first calculating the radius of interaction (*R*_a_) using the equation
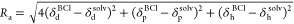
where (δ_p_^BCl^, δ_h_^BCl^, δ_d_^BCl^) and (δ_p_^solv^, δ_h_^solv^, δ_d_^solv^) are the center of the solubility sphere for BCl and the HSPs for the solvent, respectively.^51,52^ The relative energy difference (RED) is then determined using the equation
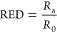
where *R*_0_ is the radius of the solubility sphere for BCl. If RED is less than 1, then the affinity between the solvent and BCl is high, and if RED is greater than 1, then the affinity between the solvent and BCl is low.^51,52^

### Nanoemulsion and Aqueous Dispersions of GNP Sample Preparation and Characterization

Oil-in-water nanoemulsions (NEs) with the oil phases (SO or Cin/CA) were prepared according to a similar procedure described previously by Zahi et al.,^53^ with slight modifications. The oil phase (1 mL) was heated to 80 °C (i.e., above *T*_gel_) and emulsified with Tween 80 (272 μL) in deionized water (9 mL) using an ultrasonic processor (UP400 St., Hielscher Inc., Wanaque, NJ, USA) operating at 13 W and 50% amplitude for 1 min at 90 °C, followed by cooling to 23 °C to give opaque, white to pale yellow homogeneous dispersions. For aqueous dispersions of GNPs, gelator **AOx24** (0–20 mg) was added to the oil phase prior to ultrasonication. For BCl-loaded nanoemulsions and aqueous dispersions of GNPs, BCl (0–25 mg) was added to the oil phase prior to ultrasonication. Finally, for BCl-loaded aqueous dispersions of GNPs with Cin/CA, BCl-saturated deionized water was used instead of deionized water. BCl-saturated deionized water was prepared by mixing BCl (250 mg) with deionized water (100 mL) with stirring at 23 °C. After 16 h, the insoluble BCl solid was separated from the BCl-saturated water phase by centrifugation (30 min at 4000 rpm) and the concentration of solubilized BCl was measured by HPLC. The solubility of BCl in deionized water was 1.39 ± 0.04 mg/mL, which is in excellent agreement with other reported values.^4^ The samples were then diluted 100–600-fold for DLS measurements, 50-fold for SEM/STEM analysis, 0–200-fold for OM analysis, and 600-fold for fluorescence microscopy analysis.

### SEM/STEM Sample Preparation

A sample of GNPs (5–10 μL) was placed on a carbon-film-coated 400 mesh copper grid (Electron Microscopy Sciences, Hatfield, PA, USA). After 10 s, the liquid was wicked away using a piece of filter paper, and the sample was allowed to dry at 23 °C for 16 h before imaging.

### High-Performance Liquid Chromatography

The quantification of BCl and CA for encapsulation efficiency (EE) calculations and solubility measurements in water and Cin was carried out using HPLC. For the analysis of samples containing only BCl, acetonitrile/water/trifluoroacetic acid (60:40:0.1) was used as the mobile phase. For samples requiring analysis of both BCl and CA, acetonitrile/water/phosphoric acid (30:70:0.4) was used. In both cases the flow rate was 1 mL/min, and BCl and CA were detected at wavelengths of 345 and 275 nm, respectively. Linear calibration ranges of 1–200 and 1–300 μg/mL were used for BCl (retention time ∼ 7 min) and CA (retention time ∼ 12 min).

### Encapsulation Efficiency

Aqueous dispersions of GNPs (1 mL) were filtered by centrifugal filtration (1690*g*, for 30 min) using Ultracel-4 cellulose membrane filters (4 mL Amicon Ultra-4, 3 kDa MWCO centrifugal filter unit, Sigma-Aldrich, Mississauga, ON, Canada). The filtered GNPs were mixed with fresh PBS solution (2 mL) and centrifuged (1690*g*, for 30 min) again, which was repeated three times to completely remove any residual BCl. The supernatants were then combined (7 mL total), and an aliquot (0.1 mL) was diluted with acetonitrile/water (0.9 mL, 60:40 v/v). The combined supernatant solution was analyzed by HPLC to quantify the amount of nonencapsulated BCl.

The encapsulation efficiency (EE) was calculated from the amount of BCl measured in the aqueous phase by HPLC (BCl_aq_) using the equations
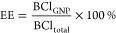

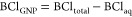
where BCl_total_ is the amount of BCl initially added and BCl_GNP_ is the amount of BCl encapsulated within the gel nanoparticles.

### Solubility Enhancement Studies

BCl (100 mg) was dispersed in Cin (1 mL) with a brief bath sonication and stirring in order to give a suspension. Varied amounts of CA (0, 25, 50, 75, 100, 150, 200, and 250 mg) were then added, and the mixtures were allowed to stir for 16 h at 23 °C. The excess BCl was separated by centrifugation, and an aliquot of the supernatant was then removed and diluted for HPLC analysis of BCl and CA.

### In Vitro Release Studies

Samples for release studies (2 mL) were first transferred to a dialysis cassette (Slid-A-Lizer G2, 20 kDa MWCO, Thermo Scientific, MA, USA), which was closed and placed in PBS (pH = 7.4, 100 mL) with stirring at 37 °C. After various time intervals, the dialysis bag was transferred into fresh PBS (100 mL) at 37 °C, and the amount of BCl released was measured by HPLC. The process was repeated until no more BCl was released. Experiments were carried out with aqueous dispersions of BCl-loaded GNPs with Cin with **AOx24** concentrations of 1, 2, and 5 wt %. The EEs for these GNP samples were 45, 44, and 42%, respectively. Control experiments were also carried out with BCl-loaded NEs and BCl powder dispersed in PBS (1.24 mg/mL).

### Cell Internalization Studies

#### BMMC Culture

All animals were sacrificed in accordance with the Canadian Council on Animal Care Guidelines and Policies (https://ccac.ca/en/about-the-ccac/) with approval from the Health Science Animal Care and Use Committee for the University of Alberta. Femurs were removed from 12 week-old C57Bl/6 mice (a kind gift from Dr. Troy Baldwin, University of Alberta) using standard dissection. Bone marrow was aspirated using a 27 gauge needle, and the cells were cultured in RPMI media (Fisher, Hampton, NH, USA) supplemented with 4 mM l-glutamine (Fisher), 50 μM β-mercaptoethanol (BME, Sigma-Aldrich, Oakville, ON, Canada), 1 mM sodium pyruvate (Fisher), 100 U/mL penicillin/100 μg/mL streptomycin (Fisher), 0.1 mM nonessential amino acids (Fisher), 25 mM HEPES (Fisher), 10% FBS (Gibco, Burlington, ON, Canada), and 30 ng/mL mouse recombinant interleukin (IL)-3 (PeproTech, Rocky Hill, New Jersey, USA), pH-7.4–7.6, in a humidified atmosphere of 5% CO_2_ in air at 37 °C. This media will be referred to as “supplemented RPMI”. The cell suspensions were maintained at a density of 0.5 × 10^6^ cells/mL for 4 weeks when the cells were tested for FcεRI and c-Kit expression by flow cytometry to confirm maturation.

#### Treatment of BMMC with BCl-Loaded NE or GNP

14 week-old BMMCs were treated with 1 μM BCl-loaded NE (0 wt % **AOx24**) or GNPs (2 wt % **AOx24**, relative to Cin) for 24 h. After 24 h, the cells were processed for flow cytometry, as described below.

#### Flow Cytometry to Measure BCl Fluorescence

To measure BCl fluorescence, BMMCs were washed three times with PBS-BSA and resuspended in 100 μL of PBS-BSA-sodium azide and analyzed with a flow cytometer. Data were generated using the FlowJo 10.6.2 software (Becton, Dickinson and Company, Franklin Lakes, NJ, USA). A healthy cell population having relatively high side scatter (indicative of cell granularity) and forward scatter (indicative of cell size) was selected and analyzed for BCl internalization to preclude the possibility of false positive fluorescence emitted by dead cells/debris having a low side scatter and forward scatter.

## Results and Discussion

**Figure 1:**
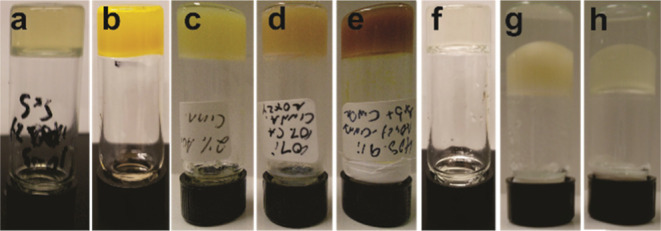
Organogels from **AOx24**. Organogels of **AOx24** (a) with SO (1 wt %), (b) with SO (2 wt %) + BCl (10 mg/mL), (c) with Cin (2 wt %), (d) with Cin (2 wt %) + CA (10 wt %), (e) with Cin (2 wt %) + CA (10 wt %) + BCl (2.5 wt %), (f) with Tw80 (2 wt %), (g) with Cin/water (1:1) (2 wt %), and (h) with SO/water (1:1) (2 wt %).

The first step in the preparation of the aqueous dispersions of GNPs was the selection of a LMWG and the gelation of various organic liquids by the LMWG. Among a variety of supramolecular organogelators available in our laboratories, the compound 2-decyltetradecanoxalamide (**AOx24**, Figure S1) was selected for the formation of aqueous dispersions of GNPs for this work. The compound **AOx24** is a versatile and efficient organogelator that forms gels with a large number of protic and aprotic organic liquids with different polarities at low gelator concentrations.^48^ Among these were organogels from **AOx24** with the water immiscible and GRAS liquids SO and Cin (Figure 1).^48^ For this work, the CGC or the minimum concentration was determined to be slightly lower for the organogels with SO (0.4 wt %) than Cin (1.6 wt %) for relatively short gel times (i.e., 30 min). Gels from **AOx24** with Cin did form at lower concentrations; however, longer gel times were required, i.e., 3 h at 1.2 wt % and 1 h at 1.4 wt %. Based on these results, gelator concentrations of 1–2 wt % should be suitable for the formation of GNPs.

The thermal stability of the organogels of **AOx24** with SO and Cin was assessed from the gel-to-solution transition temperatures (*T*_gel_) measured by (1) the inverted vial method, (2) DSC (Figure S2), and (3) rheology temperature sweeps (Figure 3c,d). Gels from **AOx24** with SO were examined at 1 and 2 wt %, while gels with Cin were examined only at 2 wt %. For the gels with SO at 1 wt %, *T*_gel_s were measured to be 61, 70, and 63 °C by (1), (2) and (3), respectively, while at 2 wt %, *T*_gel_s were 74, 70, and 60 °C by methods (1), (2) and (3), respectively (Table 1). For the gel of **AOx24** with Cin at 2 wt %, *T*_gel_s were 63, 46, and 50 °C by methods (1), (2) and (3), respectively (Table 1). The *T*_gel_s values from the rheology experiments are taken as the true *T*_gel_ values as the rheological value represents the temperature at which the elastic clusters that make the bridge between the sample edges are completely broken.^54^ Furthermore, *T*_gel_ from DSC measurements typically gives the temperature at which *G*′ and *G*″ are starting to decrease and increase relative to one another, respectively, due to the detection of weakened molecular associations (i.e., H-bonds) in a material and not connectivity phenomena linked with network formation.^54^ Finally, *T*_gel_ determined from “benchtop” rheology tests (i.e., inverted vial^50^ or falling ball^55^ tests) is well-known but less accurate,^56^ often giving the gel destruction temperature rather than true *T*_gel_.^57^ Overall, the gels with SO are more thermally stable than the gels with Cin by ∼10 °C. More importantly, gels melt at relatively low temperatures (i.e., <75 °C) at which BCl should be stable and facilitate emulsification with water.

**Table 1:** Gelation Behavior of AOx24 in Various Organic Liquids^a^

		*T*_gel_ (°C)		
solvent (CGC, wt %)	[AOx24] (wt %)	vial Inversion	DSC	rheology
sesame oil (0.4)	1	61	70	63
	2	74	70	60
cinnamaldehyde (1.4)	2	63	46	50

a CGC = critical gel concentration

**Figure 2:**
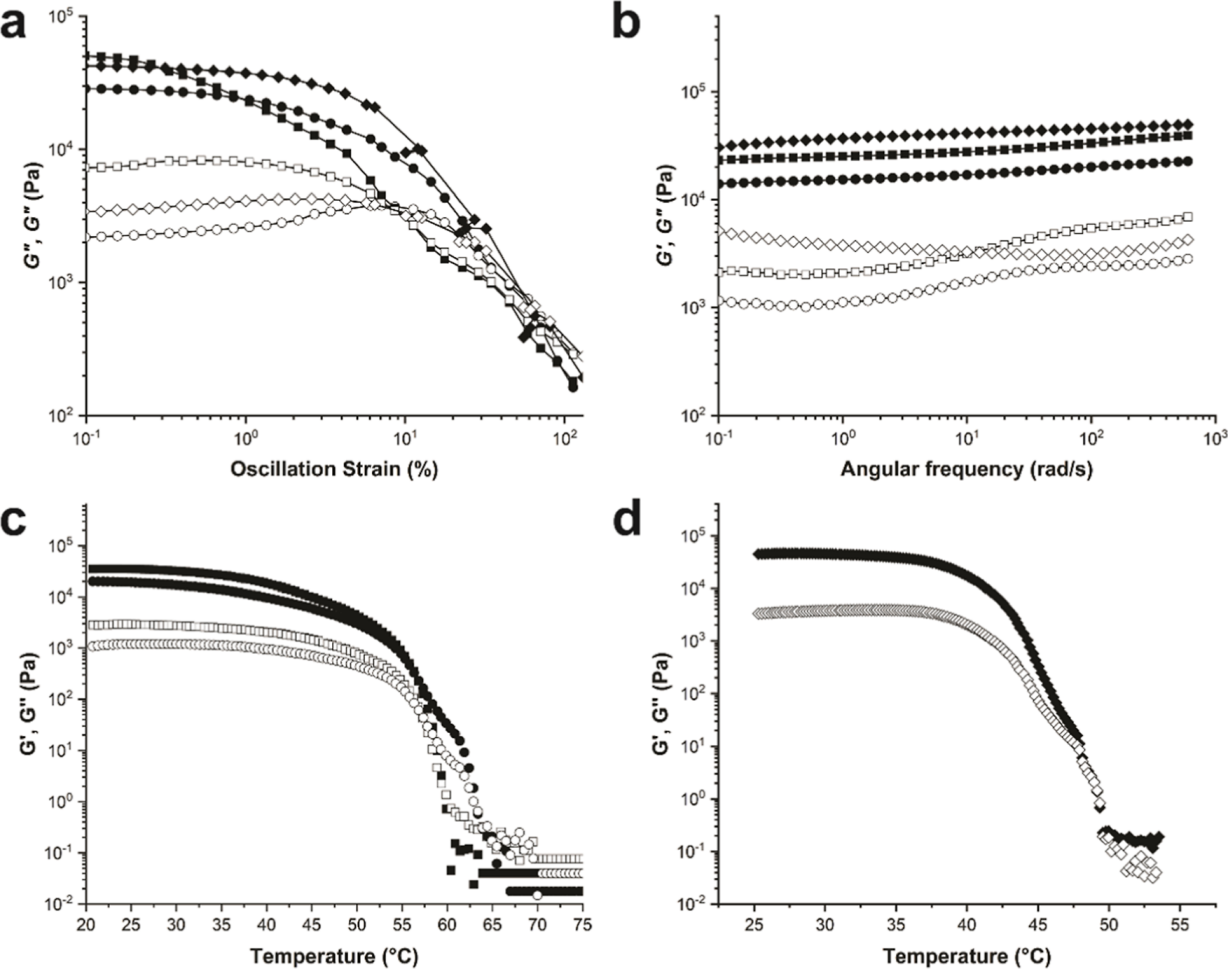
Oscillatory rheological studies of organogels of **AOx24**. (a) Frequency sweeps. (b) Amplitude sweeps. (c) Temperature sweeps for the gels of **AOx24** with SO (1 and 2 wt %). (d) Temperature sweeps for the gel of **AOx24** with Cin (2 wt %). Solid shapes = *G*′. Open shapes = *G*″. Circles = SO (1 wt %). Squares = SO (2 wt %). Diamonds = Cin (2 wt %). SO = sesame oil. BCl = berberine chloride. Cin = cinnamaldehyde.

**Figure 3:**
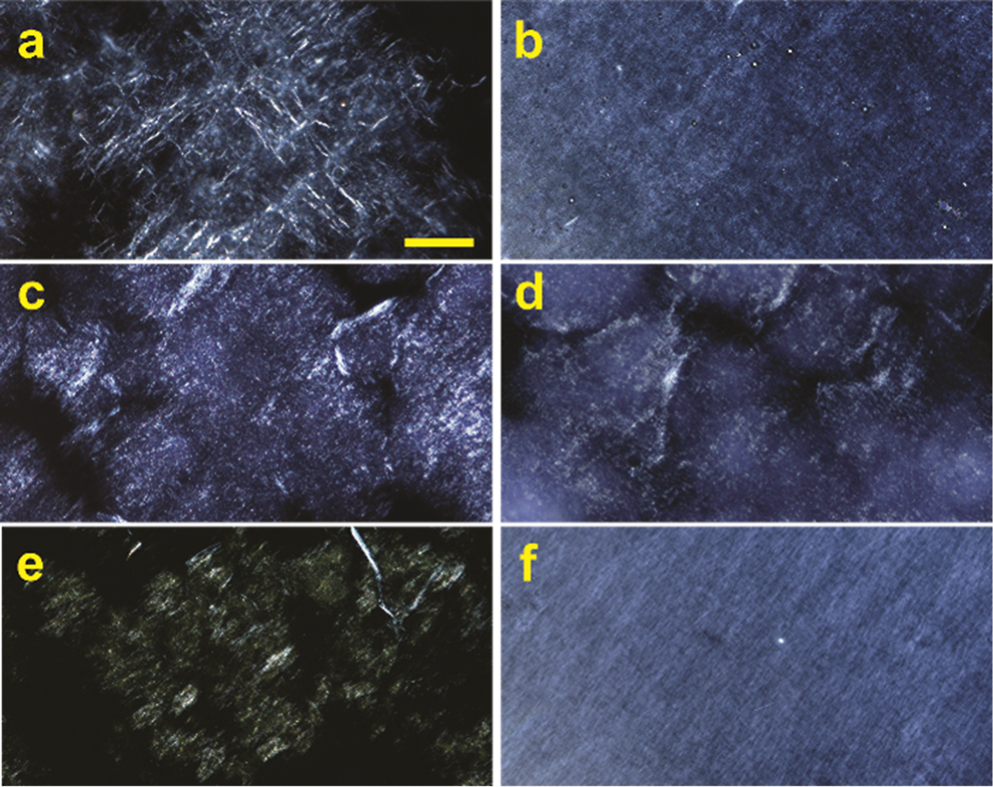
POM images of gels of **AOx24**. (a) SO (1 wt %). (b) SO (1 wt %) from SO/water (1:1). (c) Cin (2 wt %). (d) Cin (2 wt %) from Cin/water (1:1). (e) Cin (2 wt %) with BCl (1 wt %). (f) Tw80 (1 wt %). All images are 200×. The scale bar is 100 μm.

The rheological properties of gels are very important in characterizing the viscoelastic behavior and determining if the mechanical properties are suitable for specific applications. Therefore, oscillatory rheology testing was carried out on gels of **AOx24** with SO and Cin in order to examine the viscoelastic behavior, thermal stability, and mechanical properties of the constituent gel networks. First, strain sweep experiments were carried out to determine the linear viscoelastic regions (LVRs), which are defined by the region where the elastic modulus *G*′ is relatively constant and is larger than the viscous modulus *G*″. The LVR for the gel with Cin was larger than that for the gel with SO at 2 wt % from the upper strain limits ∼6% to ∼0.2%, respectively, at which *G*′ starts to decrease with increasing strain, indicating that the gel network starts to break down (Figure 2a). The crossover strains (*G*′ = *G*″) occurred at ∼9% for SO and ∼70% for Cin, respectively, which reveals the strains at which the samples are no longer gels. The values clearly show that the gel with Cin is the mechanically stronger of the two (7-fold), despite the slightly lower thermal stability. Frequency sweeps revealed a dominant elastic over viscous character, which is characteristic of a gel over the measured frequency range, as determined by the magnitude of *G*′, which is several times higher than *G*′′ (Figure 2b). At 2 wt %, *G*′ was similar (∼4000 kPa) for both gels with Cin and SO, indicating similar stiffness.

Before synthesizing aqueous dispersions of GNPs, inverted vial gel tests were carried out in the presence of various other GNP formulation components (i.e., BCl, Tw80, CA, and water) in order to confirm that the components do not interfere with the self-assembly of the gel networks from **AOx24**. Especially Tw80, CA, and water, which possess both hydrogen bond accepting and donating groups, could disrupt the intermolecular hydrogen bonding of **AOx24** molecules. Interestingly, **AOx24** formed a gel with Tw80 (Figure 1f) at 2 wt %, which is not all that surprising since the structure of Tw80 has three oxyethylene groups and considering that **AOx24** is also a good gelator for the oxyethylene solvents ethylene and propylene glycol.^48^ Gelation tests carried out in the presence of BCl in both SO and Cin confirmed that BCl does not significantly disrupt the gel networks formed by **AOx24** (Figure 1b,e). While CA does possess a carboxylic acid group that can potentially interfere with hydrogen bonding between **AOx24** molecules, gel tests with **AOx24** in Cin with CA also confirmed that CA does not significantly interfere with the self-assembly process (Figure 1d,e). Finally, water is the major component in the GNP formulations and can also act as a good hydrogen bond donor and acceptor. Phase-selective gelation experiments carried out with 1:1 SO/water and Cin/water resulted in gelled-oil phases that passed the vial inversion test and were strong enough to hold the weight of the water phase (Figure 1g,h, respectively). Clearly, water also does not disrupt the hydrogen-bonded **AOx24** molecules that form the gel networks in SO and Cin. The results of these bulk phase gelation experiments provide strong evidence that **AOx24** should be able to gel nanomicron-sized droplets dispersed in water in the presence of Tw80, CA, and BCl.

POM was used to examine the morphology of the organogels from **AOx24** SO, Cin, and Tw80 (Figure 3). Birefringent entangled masses of fibrous aggregates are observed in all six images for the organogels from **AOx24** with SO (1 wt %, Figure 3a), SO (1 wt %) from a 1:1 SO/water mixture (Figure 3b), Cin (2 wt %, Figure 3c), Cin (2 wt %) from 1:1 Cin/water (Figure 3d), Cin (2 wt %) with BCl (1 wt %, Figure 3e), and Tw80 (1 wt %, Figure 3f). Although individual fibers are difficult to discern within bundles and in densely entangled regions at the magnifications shown in Figure 3, the fibers are estimated to have widths in the 0.1–1 μm range and lengths in the 100–1000 μm range. These results indicate that the compound **AOx24** undergoes hierarchical self-assembly to form fibrous structures that entangle to form 3D SAFINs, which leads to the formation of stable organogels (Figure 1) through the entrapment of the SO, Cin, and Tw80 solvent molecules as well as BCl and trace water molecules, which is also consistent with the self-assembly of **AOx24** and other *N*-alkylated primary oxalamides in other organic solvents.^48,58^ Note that the corresponding xerogels were not examined at higher magnification by SEM because SO, Cin, and Tw80 are all nonvolatile and hindered sample preparations. These results confirm that **AOx24** should be able to form networks of submicron-scale fibers within the dispersed oil droplets, which should provide surfaces for BCl to adsorb to via weak, noncovalent interactions and/or provide a tortuous path to hinder diffusion within and egress from the oil phase.

**Figure 4:**
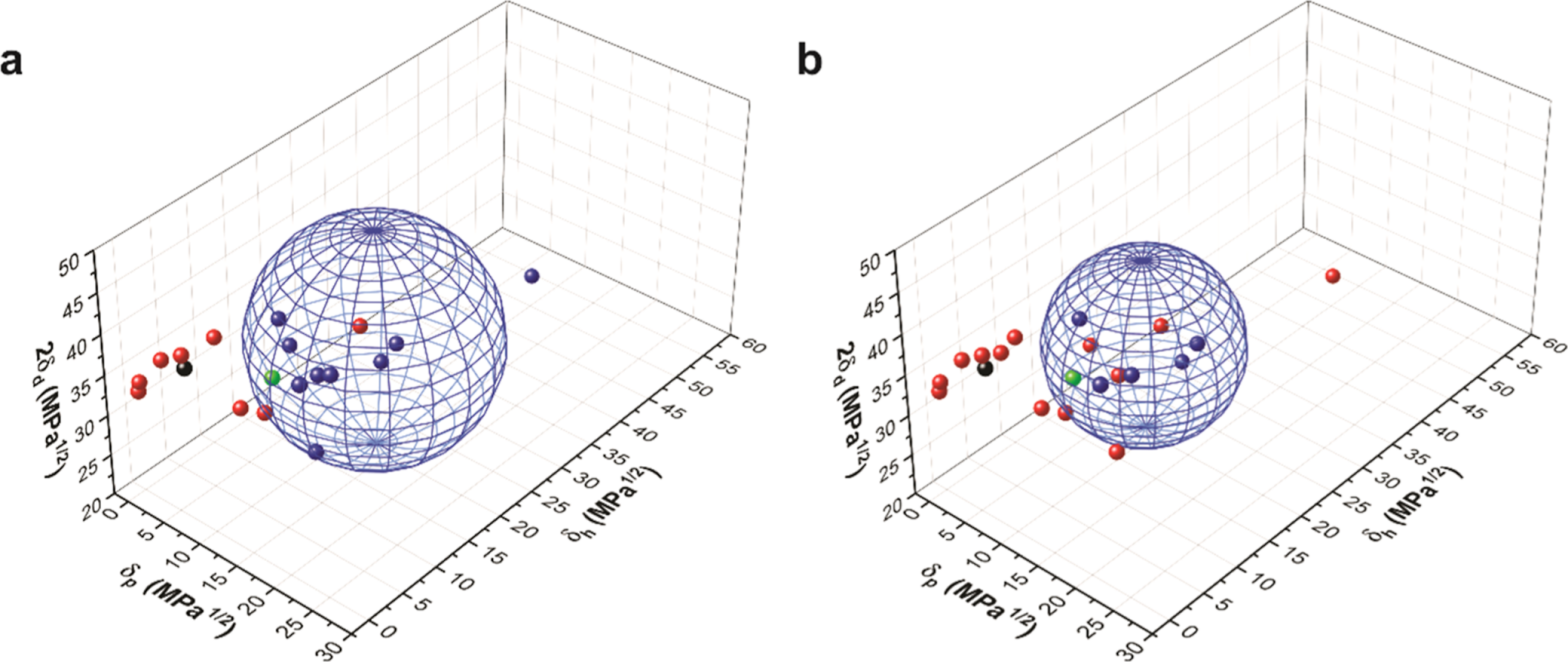
Solubility data and solubility sphere for BCl presented in Hansen space. (a) 1 mg/mL. (b) 5 mg/mL. Red = insoluble. Blue = soluble. Black = sesame oil. Green = cinnamaldehyde.

Another important criterion for the selection of SO and Cin as the oil phases for aqueous dispersions of GNPs in this study was the solubility of BCl in SO and Cin. SO was first selected based on the reported solubility of BCl in SO (2.58 mg/mL),^59^ which is low but, to the best of our knowledge, is higher than the limited reported solubility data available for BCl with water-immiscible organic solvents. The second oil phase used in this study, Cin, was found with the help of HSPs.^51,52^ Solubility tests were carried out for BCl in 19 different relatively safe solvents at 1 and 5 mg/mL (Table S1), and the data was fit to solubility spheres (Figure 4) using the commercial HSPiP software.^51,52^ From these data, the HSPs for BCl were estimated from the origin and radii (*R*_0_) of the spheres at both concentrations of 1 and 5 mg/mL (Table S2). At 1 mg/mL, the solubility sphere for BCl was determined to have HSPs of δ_d_ = 18.90 ± 0.17 MPa^1/2^, δ_p_ = 16.81 ± 0.56 MPa^1/2^, δ_h_ = 16.29 ± 0.56 MPa^1/2^, and *R*_0_ = 12.99 ± 0.49 MPa^1/2^, while at 5 mg/mL, the HSPs were δ_d_ = 19.08 ± 0.43 MPa^1/2^, δ_p_ = 19.08 ± 0.87 MPa^1/2^, δ_h_ = 13.22 ± 0.43 MPa^1/2^, and *R*_0_ = 10.12 ± 0.38 MPa^1/2^. Figure 4 shows the blue solubility sphere calculated for BCl in 3D Hansen space as well as the data points for each solvent tested in this study based on HSP values from the literature (Table S1). Using these results and the database of known HSPs for approximately ten thousand common solvents,^51,52^ the GRAS essential oil *trans*-Cin (green) was identified as a potential good solvent for BCl, which lies just within the solubility sphere (Figure 4b).

Aqueous dispersions of GNPs were prepared in two steps using a modified version of the procedure reported by Zahi et al.^53^ First, the organogels of **AOx24** with SO or Cin were heated above *T*_gel_ and emulsified in water by ultrasonication in the presence of the stabilizing agent Tw80, which is a nonionic surfactant consisting of a hydrophilic polyethoxylated sorbitan headgroup attached to a hydrophobic oleic acid tailgroup (Figure S1). When mixed with oil/water mixtures, Tw80 molecules associate with the surface of the dispersed oil phase droplets. The oleophilic/hydrophobic oleyl tail group associates with the oil phase, and the oxyethylene chains of the headgroup in the aqueous phase provide steric stabilization, inhibiting Ostwald ripening of the dispersed oil droplets. After emulsification, the emulsion was cooled below *T*_gel_ in order to commence gelation and transform the oil droplets into GNPs. NE (without gelator **AOx24**) and GNP formulations were prepared with both SO and Cin, both with and without BCl.

The mean particle hydrodynamic diameter (*D*_h_) and polydispersity index (PDI) for NEs and GNPs with SO and Cin were measured using DLS. For the formulations with SO, *D*_h_ ranged between 170 and 200 nm with acceptable PDI values of ∼0.2. Neither the gelator nor BCl had a significant effect on *D*_h_. For the formulations with Cin as the oil phase, the *z*-average *D*_h_ was smaller than for the SO formulations, ranging between 130 and 150 nm with satisfactory PDI values between 0.2 and 0.4. Again, the concentration of the gelator and the presence/absence of BCl or CA were not found to have a significant effect on the average *D*_h_. Zeta potential measurements revealed a small positive surface charge (between 0 and +5 mV) for the BCl-loaded NEs/GNPs and a slight negative surface charge (−5 and −10 mV) for the formulations without BCl. The aqueous dispersions were reasonably stable over long periods of time. After five months, the *D*_h_ values did not change significantly (140–150 nm). However, after 18 months, the *D*_h_ values for the NE and GNPs without BCl increased to between 200 and 230 nm (PDI = 0.1–0.2), while the zeta potential became more negative (−15 mV). The *D*_h_ for BCl-loaded NE and GNPs increased more significantly to between 460 and 520 nm (PDI = 0.7–0.8), while the surface charge was also negligible (i.e., 0 to −1.5 mV).

**Figure 5:**
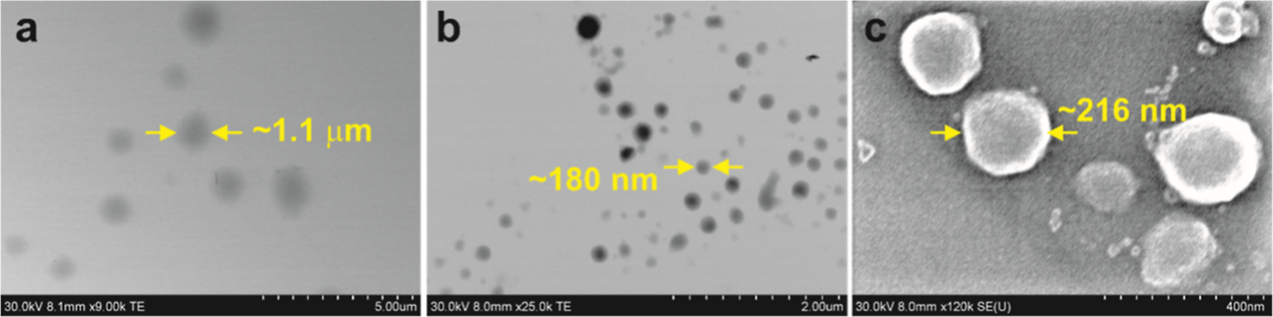
STEM and SEM images of **AOx24**-Tw80-Cin GNPs. STEM images of a sample diluted (a) 10-fold and (b) 50-fold. (c) SEM image of a sample diluted 50-fold.

The morphology of the GNPs was examined by using OM (Figures S3 and S4), fluorescence microscopy (Figure S5), SEM, and STEM (Figure 5). The smaller population of larger GNPs that were >1 μm in diameter were clearly observed in OM and fluorescence microscopy images (Figure S5), while SEM/STEM imaging was required to observe smaller nanoparticles with the average sizes measured by DLS data (Figure 5). Figure 5 shows representative SEM/STEM images of the GNPs from **AOx24** with Cin. The images clearly show different-sized populations of circular objects ranging in size from <100 nm up to several μm in diameter. The presence of these circular objects suggests a (pseudo) spherical shape and confirms that the integrity is maintained reasonably well after drying. Finally, fluorescence microscopy images of the BCl-loaded GNPs with SO confirmed the encapsulation of fluorescent BCl within the nanoparticle (Figure S5a). **AOx24**, Tw80, or SO are not fluorescent, which indicates that the fluorescence observed is due only to the fluorescently encapsulated BCl.

The EE values for the GNP formulations were determined from the amount of free BCl measured in the aqueous phase using high-performance liquid chromatography (HPLC). For the GNP formulations with SO, the EE was measured to be only ∼20%, at an initial BCl loading of 10 mg/mL (relative to the volume of SO). The low EE is due to the poor solubility (essentially insoluble) that we found for the commercial batches of BCl and SO used in this study, which was in disagreement with the reported solubility data.^59^ This discrepancy with the literature may stem from different commercial batches of BCl and SO used in the two studies. Nevertheless, the EE of ∼20% seemed reasonable, considering the poor solubility of BCl in the SO used here. In addition, the poor solubility found for BCl in SO for this work is consistent with the HSP data, which clearly shows that SO (black) lies outside of the BCl solubility spheres at both 1 and 5 mg/mL (Figure 4a).

**Figure 6:**
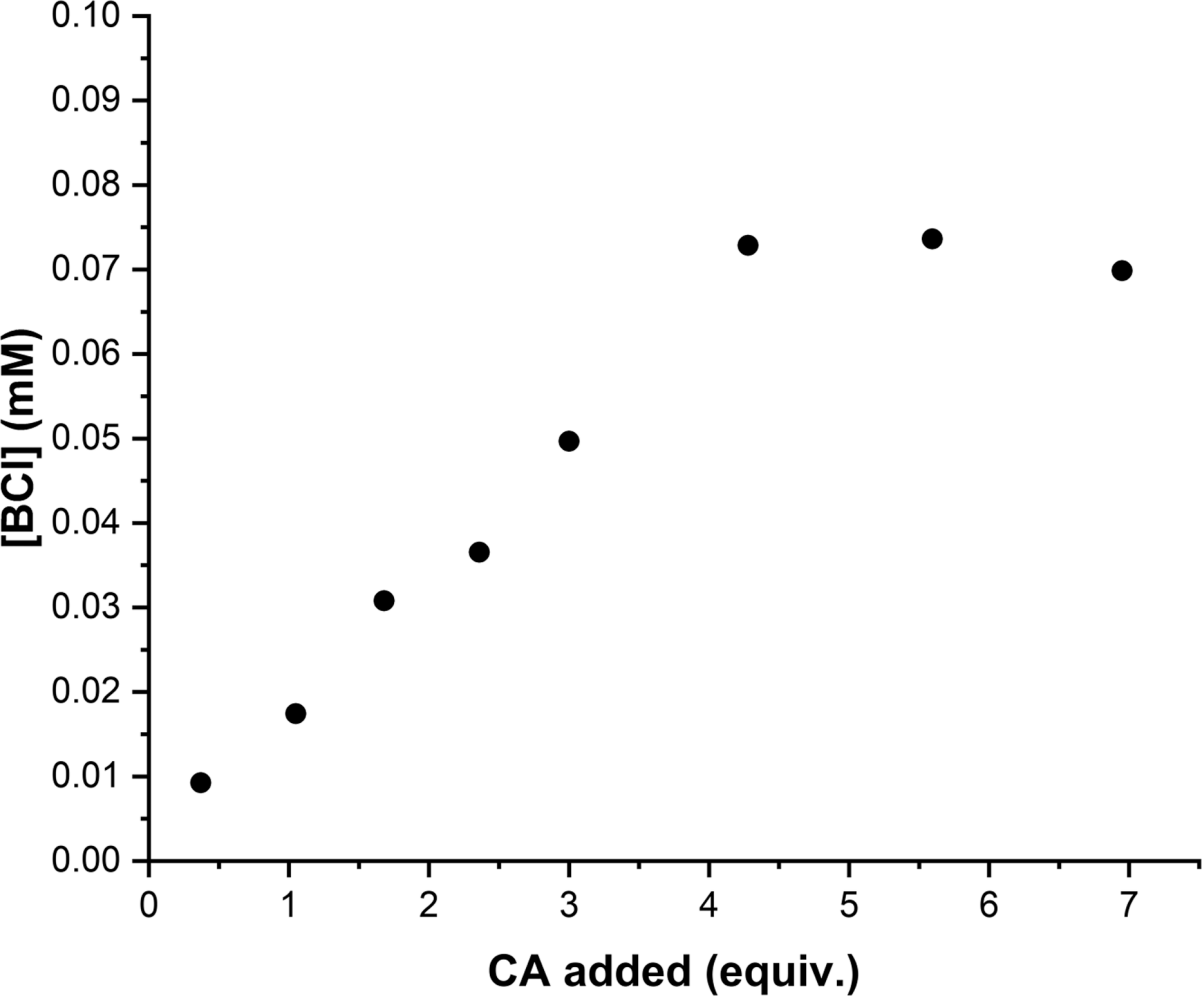
Influence of CA on the solubility of BCl in Cin at 23 °C.

The EEs for the GNP formulations with Cin with 1 and 2 wt % of **AOx24** (relative to Cin) at an initial BCl loading of 10 mg/mL were measured to be 55 and 56%, respectively. Thus, the loading capacity within the GNPs was ∼5.6 mg/mL of Cin. However, the solubility of BCl in Cin was found to vary depending on the supplier, grade, batch, and bottle size and age (after opening). This was determined to be due to the presence of variable trace concentrations of CA, which form upon oxidation of Cin in air. To exploit this interesting and unexpected finding in optimizing BCl solubility in Cin, the solubility of BCl in Cin was measured with increasing amounts of added CA. Figure 6 clearly shows that the solubility of BCl increases with increasing CA, reaching at least a 7-fold enhancement in solubility with up to ∼4 equiv of CA.

The solubility enhancement in Figure 6 suggests that both components interact to form a molecular complex. Complexation of drugs with biomolecules or other chemical agents, including metal ions, neutral organic molecules, and inclusion complexes with larger molecular host molecules, is a common strategy used to improve drug solubility.^60–62^ Complexes of BCl with several aromatic carboxylic acids are known,^63–66^ including berberine with CA.^67^ A 2:2 berberine/CA complex has been reported to form in water-DMSO mixtures in the presence of NaOH.^67^ Single-crystal X-ray analysis revealed a 2:2 berberine-CA complex in which the berberine and CA molecules are bound via hydrogen bonds with four included water molecules.^67^ However, these conditions are considerably different from those in the present work. Here, CA is likely associated with BCl via OH–Cl^–^ interactions, as observed for several other related complexes of BCl with other small-molecule carboxylic acids.^63–66^ In addition, the combination of BCl and Cin and their synergistic effects for therapeutic uses have been well documented in traditional Chinese medicines,^68^ acne treatment,^10^ additives for the prevention of pathogen biofilms on foods,^69^ anticancer drugs for lung tumors,^70^ and antidiabetic effects.^71^

Finally, the loading of BCl into the GNPs was maximized by carrying out a hot emulsification process with BCl-saturated water (∼1.3 mg/mL) instead of pure water. With the solubility limit of BCl in water already reached, no BCl should leach out from the Cin oil phase during mixing of the hot emulsion. When hot solutions of **AOx24** (0–2 wt %), BCl (25 mg), and CA in Cin (1 mL) were emulsified with BCl-saturated water (9 mL) and Tw80, followed by cooling, BCl-loaded GNPs were formed with a loading of 25 mg/mL. No change in the BCl concentration in the aqueous phase before and after emulsification was measured by HPLC, which confirmed that the loading of BCl in the GNPs did not change (25 mg/mL). At this loading, the total amount of bound BCl was only ∼65 and ∼35% in the BCl-saturated aqueous phase. The EE is essentially 100%, considering that the amount of BCl in the Cin oil phase did not change before or after GNP formation. Note that loadings greater than 25 mg/mL can be achieved with heating (i.e., 100 °C); however, precipitation/crystallization of BCl occurs over time upon standing at 23 °C over several hours to days, which would not be undesirable since this would occur only within the gelled Cin oil phase.

The cumulative release of BCl into PBS (pH = 7.4) at 37 °C was examined for BCl-loaded GNP formulations with different **AOx24** concentrations (i.e., 1, 2, and 5 wt % based on the Cin oil phase), BCl solution, and BCl-loaded NE using the dialysis membrane method^72^ in phosphate buffered saline (PBS) at pH 7.4 at 37 °C to simulate human physiological conditions. Using this method, the total release profile is governed by the rate of drug release from the carrier and the rate of permeation through the dialysis membrane. The amount of BCl released was calculated as a percentage of the total amount of BCl encapsulated within the GNPs and the BCl-saturated aqueous phase. Zero-order, first-order, Higuchi, and Korsmeyer–Peppas kinetic models^72^ were used to fit the experimental data obtained to describe the BCl release from the GNPs. The Akaike information criterion (AIC) was used in order to assess which model presents the best fit, where a lower AIC score suggests a better fit.

**Figure 7:**
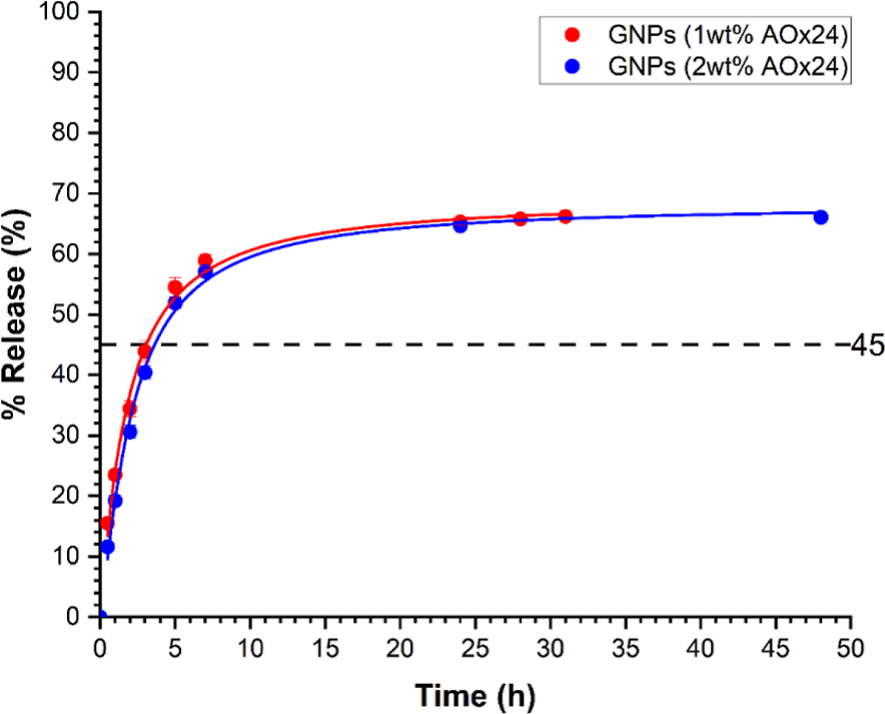
Release profiles of BCl from BCl-loaded GNPs with Cin/CA (1 and 2 wt % **AOx24**) in PBS at 37 °C. The dashed horizontal line indicates the % of BCl released from the BCl-saturated dispersed aqueous phase. Error bars represent standard deviation (*n* = 3).

Figure 7 shows the BCl release profile in GNPs prepared at two different gelator concentrations of 1 and 2 wt % (relative to the oil phase Cin) over 48 h. For these experiments, 55% of BCl was encapsulated in the GNPs, while 45% of BCl was in the aqueous carrier phase solution. The GNP release profiles at 1 and 2 wt % **AOx24** demonstrated a gradual, sustained release of BCl from the GNPs up to ∼60% after 7 h, which did not change significantly up to 24 h or more. Since the initial aqueous carrier phase contained 45% of the total BCl, these results indicated that only ∼15% of the encapsulated BCl was released. The cumulative drug release was best fit by the Korsmeyer–Peppas model,^72,73^ according to highest regression coefficient (0.99) and lowest AIC value (0.5 to −25.4, Table S3). The Korsmeyer–Peppas rate constant (*k*_KP_) was determined to be ∼20 with an exponent *n* of ∼0.55 (Table S3), which indicates that the release of BCl from the GNPs occurs via non-Fickian diffusion (anomalous transport), i.e., where more than one type of phenomenon of drug release is involved (i.e., diffusion, swelling, osmosis, partitioning, and erosion/degradation).^74^ The release of BCl from the GNPs here is likely a multistep process, possibly involving (1) association/dissociation of BCl from the surface of the gel network of aggregated **AOx24**, (2) diffusion of BCl through the oil phase via a tortuous path provided by the gel network, (3) partitioning across the oil–water interface, governed by the oil–water partition coefficient as well as a possible barrier provided by the Tw80 molecules at the interface, and (4) diffusion of BCl or BCl-loaded GNPs through the aqueous donor media to the dialysis membrane, before (5) permeation across the dialysis membrane into the recipient media.

In order to gain insight into the rate of permeation across the dialysis membrane, a control experiment involving the release of BCl from an aqueous donor solution (∼1.3 mg/mL) was examined. The release profile is shown in Figure S6, which demonstrated a gradual, sustained release of BCl from the GNPs up to ∼70% after 6 h. Beyond 6 h, the release rate slowed considerably, and after 24 h or more, nearly all of the BCl was released into the recipient media. The cumulative BCl release data for BCl solution was best fitted to a first-order kinetic model with a regression coefficient of 0.99, an AIC value of −31 (Table S3), and a rate constant of 0.04 h^–1^ (Table S3). These results indicate that the permeation of BCl through the dialysis membrane is relatively slow and is nearly complete after 24 h (>90%).

Finally, the release profile of BCl from BCl-loaded NE (0 wt % **AOx24**) is shown in Figure S6. The BCl-loaded NE demonstrated a gradual sustained release of BCl up to ∼60% after 3 h, which leveled off at ∼80% after 7 h and did not change further after 24 h (∼80%). The cumulative release from the NE best followed a first-order release kinetic model with a regression coefficient of 0.99, an AIC value of −25, and a rate constant comparable to that of the BCl solution (*k*_1_ = 0.05 h^–1^, Table S3). Clearly, the release of BCl from the NE differs from that of the GNPs, which strongly suggests that the gel network from **AOx24** forms within the NE droplets and affects the BCl release rate either via weak interactions between BCl and the gel network or the presence of a tortuous path that hinders diffusion within and egress from the oil phase.

**Figure 8:**
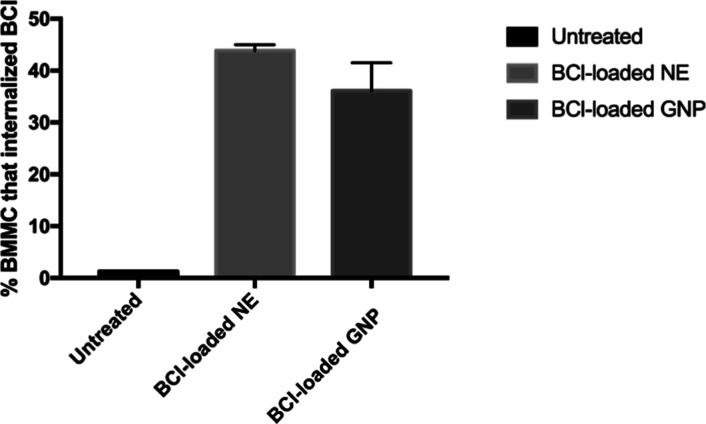
Internalization efficiency of BCl from BCl-loaded nanoemulsions (NE) and gelled-oil nanoparticles (GNPs) from **AOx24** (2 wt %) with Cin/CA in PBS. BMMCs were treated with 1 μM BCl-loaded NE or GNPs for 24 h followed by flow cytometry to determine % BMMC that internalized BCl. Ut represents untreated BMMCs that were treated with an equal amount of PBS.

Recently, the effect of **AOx24** on the viability of mouse BMMCs was reported, which suggested that **AOx24** was not cytotoxic up to 10 μM and could be used for biomedical applications.^48^ For this work, the effect of **AOx24** on the internalization efficiency of BCl into BMMC was examined using flow cytometry by comparing the effects of BCl-loaded NE versus GNPs from **AOx24** (2 wt %). Figure 8 shows that after 24 h, a significant proportion of healthy BMMCs (∼36–44%) internalized BCl from both formulations, which suggests that internalization, followed by the release of BCl from BCl-loaded NE or GNP is comparable and that the gelator **AOx24** does not interfere with internalization efficiency of BCl into BMMCs. Therefore, these results suggest that the GNPs could potentially be utilized as a drug delivery vehicle.

## Conclusions

In summary, aqueous dispersions of GNPs have been successfully developed for the encapsulation and oral delivery of BCl using a LWMG based on the N-alkylated primary oxalamide LMWG, **AOx24**. The properties of the organogels from **AOx24** with two GRAS oil phases, SO and Cin, were characterized in order to confirm their suitability for the formation of GNPs and the encapsulation of BCl. Initial GNP formulations using SO gave GNPs with low EE and loading of BCl, which was significantly improved using the oil phase Cin instead. An unexpected result was the 7-fold improvement of the solubility of BCl in Cin via complexation with 4 equiv of CA, which enabled a significantly higher loading of BCl into the GNPs. The resulting dispersions of GNPs exhibited long-term stability over sedimentation, while in vitro release studies showed that only ∼15% of the encapsulated BCl payload was released into PBS (<48 h), demonstrating the potential for longer term persistence in the circulatory system and delivery across lipophilic barriers. Both GNP formulations are currently under investigation in our laboratories for cell cytotoxicity and bioavailability and will be reported in due course. Overall, this study demonstrated the application of aqueous dispersions of GNPs in the oral delivery of compounds with poor solubility in water and provided a promising platform for the delivery of poorly soluble nutraceuticals/therapeutics with high loading capacity and oral bioavailability for biomedical applications.

## Supporting Information Available

Chemical structures of **AOx24**, BCl, Cin, CA, and Tw80; DSC traces for organogels of **AOx24** with SO and Cin at 1 and 2 wt %; HSP study data for BCl with various solvents; optical and fluorescence microscopy images of NEs and GNPs; and release profiles and kinetic modeling data of BCl-loaded solution, NE, and GNPs (PDF)
