# Supplementary material for: Development of Gelled-Oil Nanoparticles for the Encapsulation and Release of Berberine
Source: ACS Omega. 2023 Sep 6;8(37):33774–84. doi: 10.1021/acsomega.3c04230 (PMC10515596; doi:10.1021/acsomega.3c04230)
Supplement: Supplementary file 1 — ao3c04230_si_001.pdf [file ao3c04230_si_001.pdf]

Supporting Information for

Development of Gelled-Oil Nanoparticles for the Encapsulation and Release of Berberine

Darren A Makeiff,* Brad Smith, Khalid Azyat, Mike Xia and Syed Benazir Alam

Nanotechnology Research Center, National Research Council of Canada, 11421 Saskatchewan Drive, Edmonton, Alberta, Canada, T6G 2M9, E-mail: Darren.Makeiff@nrc.ca

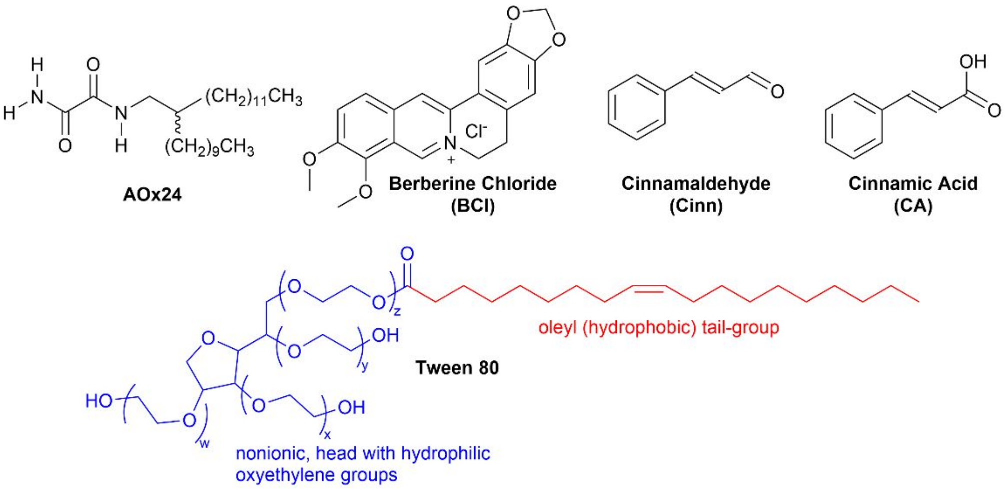

Figure S1. Chemical structures of organic compounds used in this study

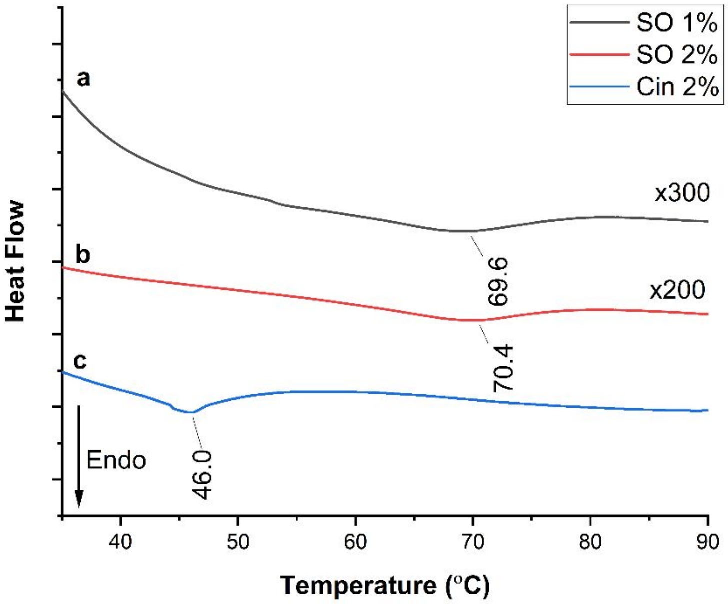

Figure S2. Differential scanning calorimetry traces for organogels of **AOx24** with sesame oil at (a) 1 wt % and (b) 2 wt % and (c) cinnamaldehyde at 2 wt %.

Table S1. Solubility test results for berberine chloride

**Solvent**	**δp ($MPa^{1/2}$)**	**δ_h ($MPa^{1/2}$)**	**$2\delta_d$ ($MPa^{1/2}$)**	**Result (1 mg/mL)**	**Result (5 mg/mL)**
Cinnamaldehyde	12.4	6.2	36.8	-	-
cis-cinnamic acid	3.9	10.6	38.2	-	-
Chloroform	3.1	5.7	35.6	-	I
1,4-Dioxane	1.8	9	35	I	I
Acetone	10.4	7	31	I	I
Chlorobenzene	4.3	2	38	I	I
Cyclohexane	0	0.2	33.6	I	I
Hexadecane	0	0	32.6	I	I
MEK	9	5.1	32	I	I
Propylene Glycol	10.4	21.3	33.6	I	I
Sesame Oil	3.5	3.09	35.38	I	I
Toluene	1.4	2	36	I	I
1-Butanol	5.7	15.8	32	S	I
Acetonitrile	18	6.1	30.6	S	I
Benzyl Alcohol	6.3	13.7	36.8	S	S
DMF	13.7	11.3	34.8	S	I
DMSO	16.4	10.2	36.8	S	S
Ethanolamine	15.5	21	34	S	S
Methanol	12.3	22.3	29.4	S	S
Propylene Carbonate	18	4.1	40	S	S
Water	16	42.3	31	S	I

All tests were carried out using 1 mL of solvent. I = insoluble. S = soluble.

Table S2. Hansen solubility parameters determined for berberine chloride (BCl) at 1 and 5 wt %

**BCl wt %**	**δ_d ($MPa^{1/2}$)**	**δ_p ($MPa^{1/2}$)**	**δ_h ($MPa^{1/2}$)**	**R ($MPa^{1/2}$)**	**Fit**
1	18.90±0.17	16.81±0.46	16.29±0.56	12.99±0.49	0.882
5	19.08±0.43	19.08±0.87	13.22±0.28	10.12±0.38	0.889

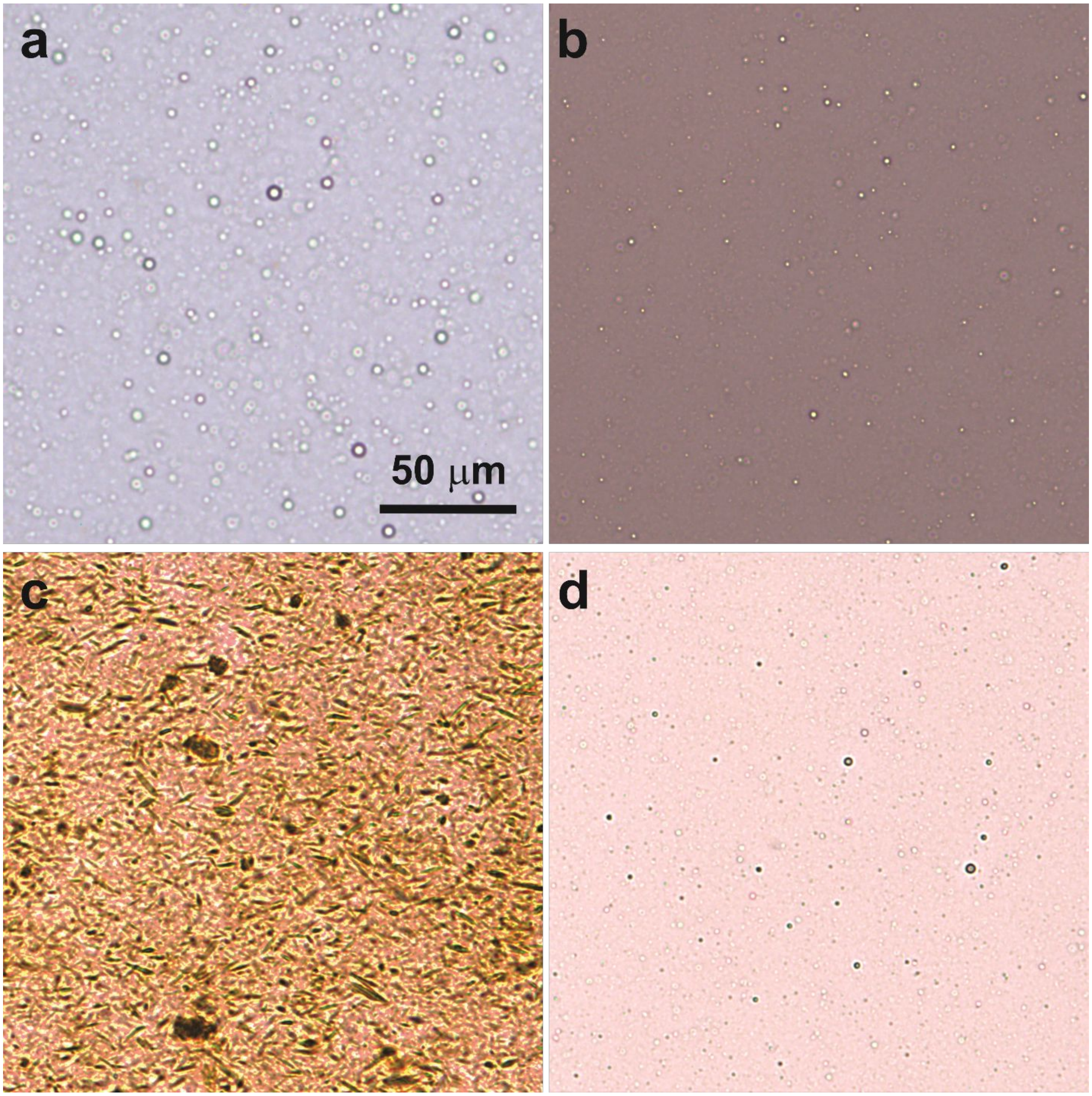

Figure S3. Optical microscopy images of sesame oil (SO) nanoemulsions (NEs) and gelled-oil nanoparticles (GONPs) from **AOx24** (1 wt %) with and without berberine chloride (BCl, 1 wt %). (a) NE. (b) GONPs. (c) and (d) BCl-loaded GONPs. (a), (b) and (c) were diluted 200-fold

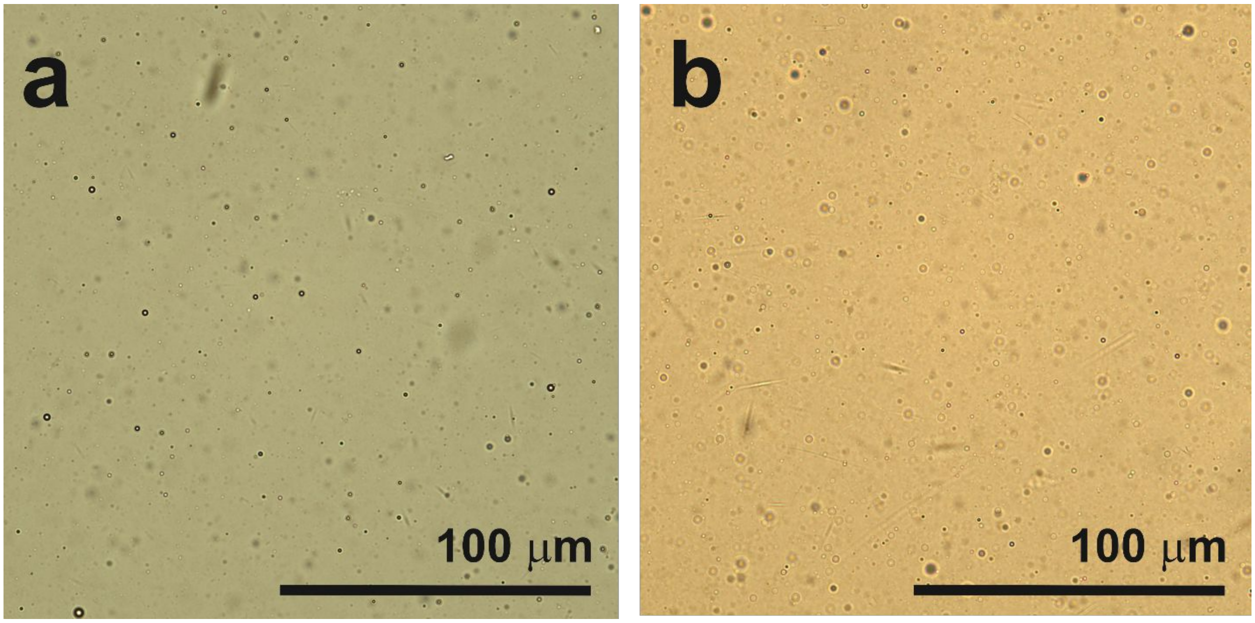

Figure S4. Optical microscopy images of gelled-oil nanoparticles (GONPs) from **AOx24** (2 wt %) with cinnamaldehyde with (a) and without (b) berberine chloride (BCl, 1 wt %) diluted 50-fold.

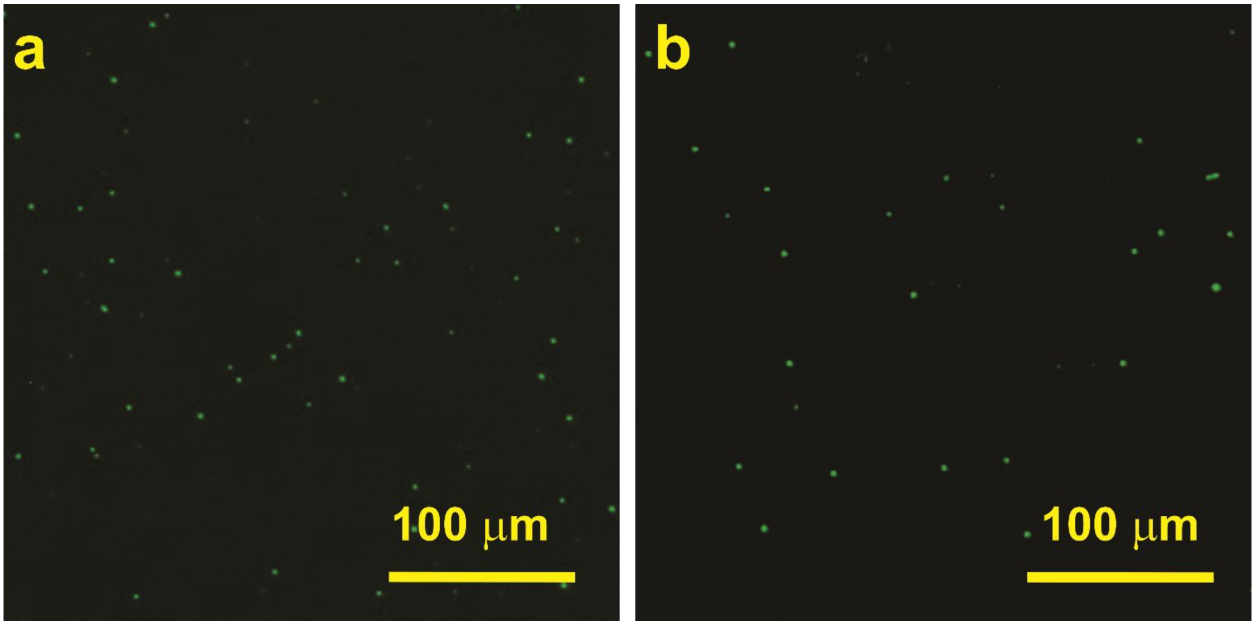

Figure S5. Fluorescence microscopy images of berberine chloride loaded (BCl, 1 wt %) gelled-oil nanoparticles (GONPs) from **AOx24** (1 wt %) with (a) sesame oil (SO) and (b) cinnamaldehyde. Diluted 600-fold.

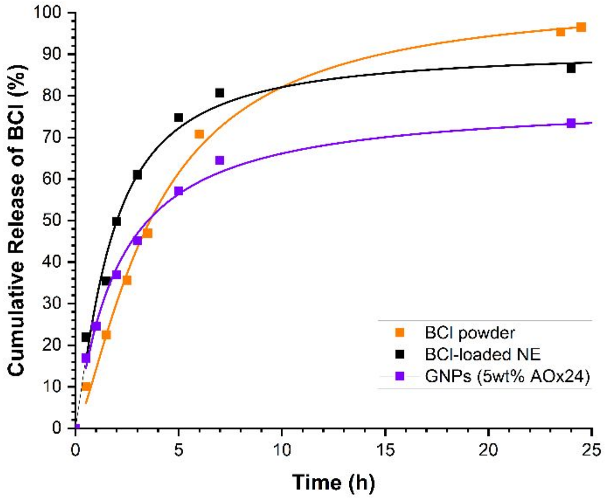

Figure S6. The release profiles of BCl from aqueous BCl solution, BCl-loaded NE and GNPs with Cin/CA (5 wt % **AOx24**) in PBS at 37°C.

Table S3. Kinetic models used to fit the data for the release of berberine chloride (BCl) from aqueous solution, nanoemulsion (NE) and gel nanoparticles (GNPs).

		Drug Release Model												
	% BCl	0 order			1st order			Higuchi			Korsmeyer-Peppas			
	release	r^2	AIC	k_0	r^2	AIC	k_1	r^2	AIC	k_H	r^2	AIC	k_{KP}	n
**Solution**	97	0.989	28.92	11.64	0.991	-31.31	0.04	0.954	37.47	29.02	0.999	-16.08	17.04	0.79
**Nanoemulsion**	90	0.951	42.44	14.23	0.985	-24.92	0.05	0.987	30.27	34.40	0.978	-5.71	31.58	0.55
**GNPs (1 wt % AOx24)**	66	0.873	41.33	7.80	0.991	29.68	5.73	0.990	23.39	23.38	0.989	0.52	23.26	0.52
**GNPs (2 wt % AOx24)**	66	0.909	38.86	7.86	0.893	-13.47	0.07	0.989	24.17	23.10	0.988	-25.44	18.94	0.62
**GNPs (5 wt % AOx24)**	75	0.999	13.74	7.80	0.990	-13.47	0.07	0.990	16.83	23.38	0.995	-33.72	23.25	0.52

r^2 = regression correlation. AIC = Akaike information criterion. k_0 = zero order release constant. k_1 = first order release constant. k_H = Higuchi model release constant. k_{KP} = Korsmeyer-Peppas model release constant. Values in green indicate the best fit.
